# Remineralizing effect of a zinc-hydroxyapatite toothpaste on enamel erosion caused by soft drinks: Ultrastructural analysis

**DOI:** 10.4317/jced.53790

**Published:** 2017-07-01

**Authors:** Marco Colombo, Maria Mirando, Davide Rattalino, Riccardo Beltrami, Marco Chiesa, Claudio Poggio

**Affiliations:** 1 Department of Clinical, Surgical, Diagnostic and Pediatric Sciences - Section of Dentistry. University of Pavia, Italy

## Abstract

**Background:**

The aim of the present *in vitro* study was to evaluate the protective effects of a zinc-hydroxyapatite toothpaste on repairing enamel erosion produced by a soft drink (Coca-Cola) compared to toothpastes with and without fluoride using Scanning Electron Microscopy (SEM).

**Material and Methods:**

Fifty specimens were assigned to 5 groups of 10 specimens each. (Group 1: no erosive challenge, no toothpaste treatment, group 2: erosive challenge, no toothpaste treatment, 3: erosive challenge, toothpaste without fluoride, group 4: erosive challenge, fluoride toothpaste treatment, group 5: erosive challenge, zinc-hydroxyapatite toothpaste treatment). Repeated erosive challenges were provided by immersing bovine enamel specimens (10 per group) in a soft drink for 2 min (6mL, room temperature) at 0, 8, 24 and 32 h. After each erosive challenge, the toothpastes were applied neat onto the surface of specimens for 3 min without brushing and removed with distilled water. Between treatments the specimens were kept in artificial saliva. The surface of each specimen was imaged by SEM.

**Results:**

Statistically significant differences were found between the samples used as control and those immersed in Coca-Cola (group 1 and 2): indeed among all groups the highest grade of damage was found in group 2. Instead the lowest grade was recorded in the samples of group 5 (Zinc hydroxyapatite toothpaste).

**Conclusions:**

The results of this study confirmed the potential benefit the Zn-HAP technology could provide in protecting enamel from erosive acid challenges. The treatment of erosively challenged enamel with Zn-Hap toothpaste showed a clear protective effect.

** Key words:**Dental erosion, enamel, SEM, toothpaste.

## Introduction

Dental erosion has been defined as pathologic, non-bacterial dental hard tissue loss induced by extrinsic or intrinsic acids or chelators acting on plaque-free tooth surfaces (1,2).

Dental erosion is a common problem in modern societies due to the increased consumption of acidic drinks, such as soft drinks, sport drinks, fruit juices, which have a high potential to cause enamel demineralization (3). Dietary changes and inadequate oral hygiene have led to enamel erosion becoming more frequent among young people.

The main acid involved in these processes is citric acid, a constituent of many fruit juices and acidic soft drinks (4,5). The typical concentration present in many acidic soft drinks is 0.2-0.004 M for the fruits juices and 0.015-0.05 M for the acidic soft drinks (5,6). The three carboxyl groups confer the high chelating properties on citric acid, which forms soluble complexes with calcium ions, enhancing the enamel dissolution to achieve saturation levels of the calcium-acid complex (5,7,8).

Dental enamel consists of 95% calcium hydroxyapatite, 4% water and about 1% organic material. Biological and chemical factors in the oral environment influence the progress of enamel softening and erosion. Saliva provides protective effects by neutralizing and clearing dietary acids; it is also a source of inorganic ions necessary for the remineralization process (9). Enamel has no spontaneous biological capability to be repaired when affected by specific dental pathologies such as caries, abrasions or fractures because it contains no cells (10). The loss of substance by erosion is a cyclic and dynamic process with periods of demineralization and remineralization. Thus, preventive measures against erosion are required.

Dental clinicians often ignore or overlook the very early stages of erosion, dismissing minor tooth surface loss as a normal and inevitable occurrence of daily life (11).

Toothpastes have been considered effective and accessible vehicles to improve enamel resistance to erosive attacks (12). Many types of toothpaste recently introduced are claimed to prevent erosion. Fluoride dentifrices have been shown to have some protective effect against the erosive challenge of a cola drink *in vitro* (13). However, conventional fluoride-containing toothpastes do not appear to be able to protect sufficiently well against erosive challenges (14). As the use of fluoride dentifrices is ubiquitous, tooth erosion prevalence is nonetheless on the rise, suggesting not all marketed fluoride toothpastes are sufficiently formulated to protect against enamel loss in the face of substantial acidic insult.

New toothpastes formulations have therefore been developed to provide more effective protection from dietary acids and hence effective protection against enamel erosion.

The aim of the present study was to test the impact of toothpaste with Zinc-Hydroxyapatite (Zn-HAP) on repairing enamel erosion compared to toothpastes with and without fluoride.

## Material and Methods

The *in vitro* study reported here utilized a cyclic demineralization/remineralization model in which bovine enamel specimens were exposed to an erosive challenge, toothpaste treatment and storage in artificial saliva at the start (0h) and after 8h, 24h and 32h.

The following three toothpastes were tested.

- toothpaste without fluoride (Subito/Incos Cosmeceutica s.r.l., 40050 Funo, Italy),

- fluoride toothpaste, 1450 ppm F- as NaF (Eufresh/CIO Farmaceutici s.r.l., 81100 Caserta, Italy),

- toothpaste with Zn-HAP (Microrepair®), without fluoride (Biorepair/Coswell S.P.A., 40050 Funo, Italy).

-Specimen preparation 

Enamel specimens were prepared from fifty freshly extracted bovine permanent mandibular incisors obtained from a local slaughterhouse (INALCA, Ospedaletto Lodigiano, Lodi, Italy). Teeth had to be free of cracks, hypoplasia and white spot lesions. After extraction, teeth were cleaned to remove soft tissue and stored in a solution of 0.1% (wt/vol) thymol. The enamel specimens were cut at the enamel-dentin junction with a high-speed diamond rotary bur with a water-air spray. The samples were placed into Teflon molds measuring 10 x 8 x 2 mm and embedded in self-curing, fast-setting acrylic resin (Rapid Repair, DeguDent GmbH, Hanau, Germany) in such a way that the exposed buccal surface was plano-parallel to the bottom of the mold.

-Experimental Groups

The study involved five different treatment groups:

group 1: no erosive challenge, no toothpaste treatment,

group 2: erosive challenge, no toothpaste treatment, 

group 3: erosive challenge, non-fluoride toothpaste treatment,

group 4: erosive challenge, fluoride toothpaste treatment,

group 5: erosive challenge, Zn-HAP toothpaste treatment.

-Erosive Challenge

A popular soft drink (Coca Cola / Coca Cola Company, Milano, Italy) was chosen for the erosive challenge. The pH at 20˚C, buffering capacity, concentration of calcium and phosphate of the beverage were measured (15). Measurements were performed in triplicate and average values calculated.

The specimens were immersed in 6mL of the soft drink for 2 min at room temperature before rinsing with deionized water. Four erosive challenges were carried out at 0, 8, 24 and 32 h, hence for a total of 8 minutes (16).

A soft drink (Coca Cola, Coca Cola Company, Milano, Italy) was chosen for the demineralization process (10). The pH at 20˚C, buffering capacity, and concentration of calcium and phosphate of the beverage were measured by standard chemical methods. The pH of soft drink was measured with a pH meter (Accumet AB15, Fisher Scientific, Pittsburgh, PA). Ca2- and PO43- were determined by flame atomic absorption (Perkin Elmer 1100 B spectrophotometer). Measurements were performed in triplicate and average values calculated ( Table 1).

**Table 1:**
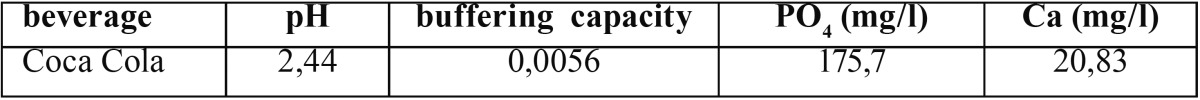
Chemical properties of the soft drink used in the study.

The samples were then assigned to the five treatment groups with 10 specimens per group.

The toothpastes were applied neat onto the surface of the specimens to cover the enamel surface without brushing and then wiped off with distilled water washing after every treatment to remove residual toothpaste; the control specimens (group 1) were taken on storage for the whole experimentation and they did not receive any treatment. The toothpastes were applied to the enamel surfaces for 3 min at 0, 8, 24 and 36 h; during these intervals, the specimens were kept in artificial saliva. The specimens of group 2, 3, 4 and 5 were immersed in 6 ml of the soft drink for 2 min at room temperature before rinsing with deionized water. Four consecutive intervals of the immersion procedure were carried out (13). The immersions in the soft drink were repeated as described above at 0, 8, 24 and 36 h.

-Toothpaste Treatment

The toothpastes were applied neat onto the surface of the specimens without brushing to cover the entire surface and removed after 3 minutes by washing with distilled water. The toothpastes were applied at 0, 8, 24 and 32 h (16) immediately after the erosive challenge.

-Specimen Storage

The enamel specimens were stored in artificial saliva (pH 7.0, 14.4 mMNaCl; 16.1 mMKCl; 0.3m mM Cl2.6H20; 2.9 mM K2HPO4; 1.0 mM CaCl2.2H2O; 0.10 g/100 ml sodium carboxymethylcellulose) (17) between the erosive challenge/ toothpaste treatment sessions.

-Scanning Electron Microscopy (SEM) 

The specimens were gently air dried, dehydrated with alcohol, sputter-coated with gold. Enamel and dentin were characterized using a field emission scanning electron microscope (FE-SEM, MIRA3, TESCAN). Serial SEM microphotographs of the surfaces of each specimen at 2.50 KX and 5.00 KX original magnifications were obtained (14). A systematic assessment method was adopted for grading the SEM images. SEM images recorded were evaluated in terms of enamel damages by three experienced assessors who randomly examined the samples twice in a blind manner. A scoring scale ( Table 2) was adopted to describe the enamel surface (18).

**Table 2:**
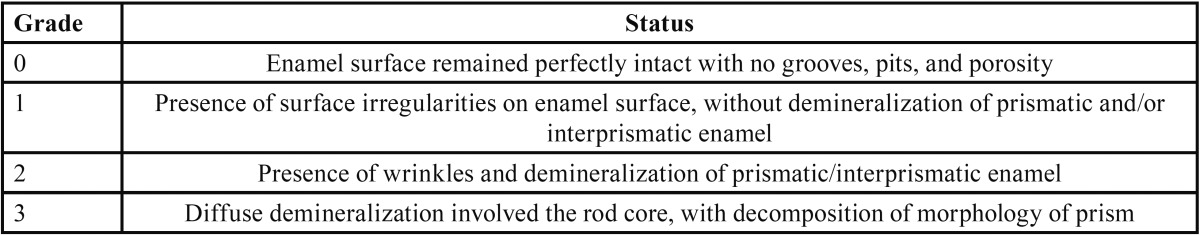
Scoring criteria used for the evaluation of SEM images.

-Statistical analyses

Descriptive statistics for the scores of the morphological analysis were calculated. Data were analyzed with the Kruskal-Wallis test. The Mann-Whitney U test was performed for post hoc comparisons. Significance was set at a *P* value <0.05. In order to check the intra - and inter-observer reliability the Intraclass Correlation Coefficient was calculated; it was greater than 0.9.

## Results

**Figure 1:**
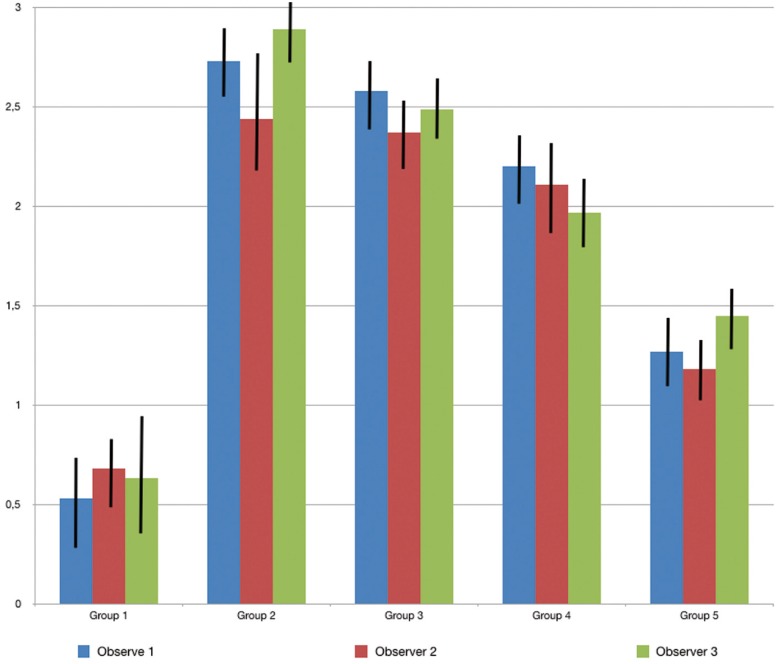
Means and standard deviations of the morphological SEM scores provided by the three observers.

**Figure 2:**
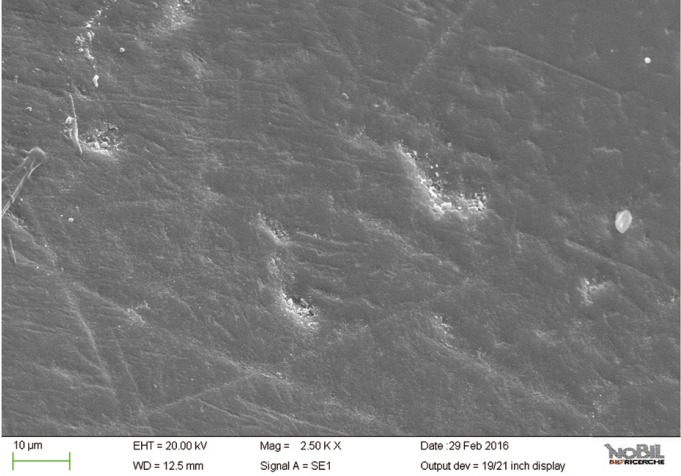
SEM image at 2.50 KX magnification of intact enamel surface (Group 1).

**Figure 3:**
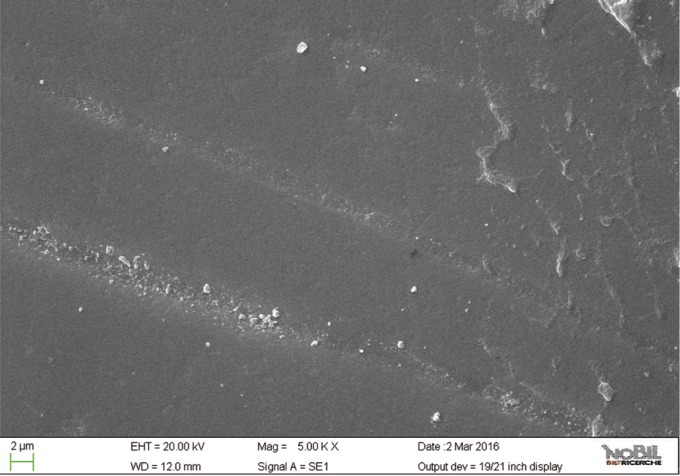
SEM image at 5.00 KX magnification of intact enamel surface (Group 1).

The mean amounts of scores for the morphological analysis of the images are reported in  Table 3 and in figure 1. The Kruskal-Wallis test showed the presence of significant differences among the different groups (*p*<0.05). On enamel surfaces not exposed to the erosive challenge by the soft drink (group 1), the typical structures of sound enamel such as grooves and perichimata lines were apparent; also small depressions or ditches or grinding marks were found indicative of the cumulative mechanical effects the teeth have experienced were observed (Figs. 2,3). The morphological scores assigned to the samples of group 1 by the three experienced assessors are overall 0.61± 0.52.

**Table 3:**
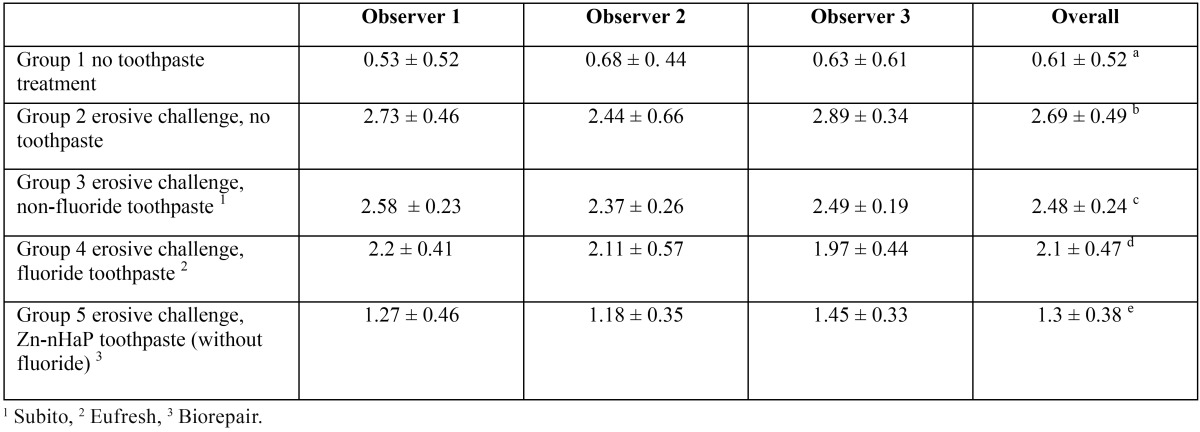
Means and standard deviations of the morphological SEM scores provided by the three observers and overall. Different superscript letters indicate significant differences (*p*< 0.05).

**Figure 4:**
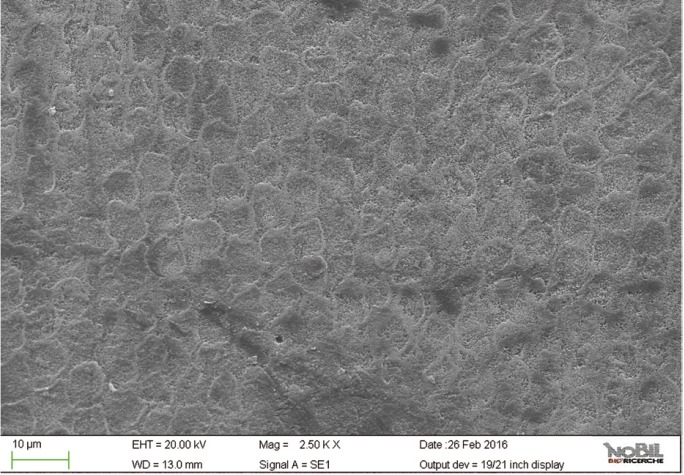
SEM image at 2.50 KX magnification of enamel exposed to Coca-Cola (Group 2).

**Figure 5:**
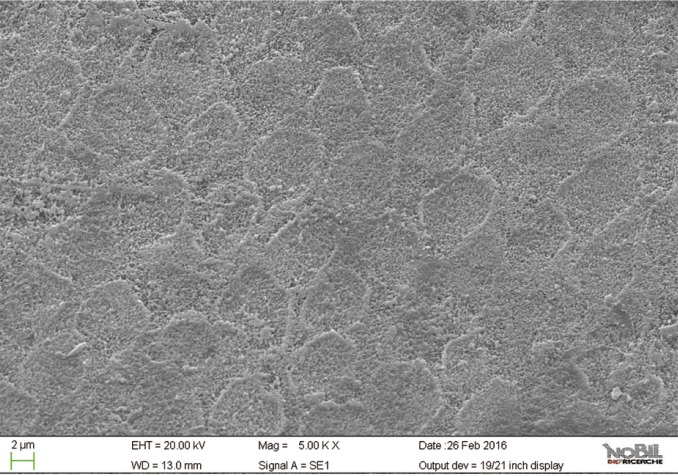
SEM image at 5.00 KX magnification of enamel exposed to Coca-Cola (Group 2).

The morphological scores for the acid challenged specimens (group 2) were significantly higher than the scores for the specimens not exposed to acid challenge. (Mann-Whitney U test, *p*<0.05). The enamel surface of teeth exposed to the acidic challenge by the soft drink clearly demonstrated deep changes in enamel structure (Figs. 4,5). After 32 min exposure to the acidic challenge (four immersions of 8 min each) an irregular pattern of surface erosion could be observed and the presence of honeycomb structures suggests demineralization of enamel prisms.

The acid-challenged specimens treated with the toothpastes with and without fluoride (groups 4 and group 3 respectively) demonstrated a lower degree of demineralization on the enamel surface if compared to group 2. The SEM images are clear and explanatory and this is reflected in the lower morphological scores for these groups compared to the acid-challenged samples not treated with toothpaste.

**Figure 6:**
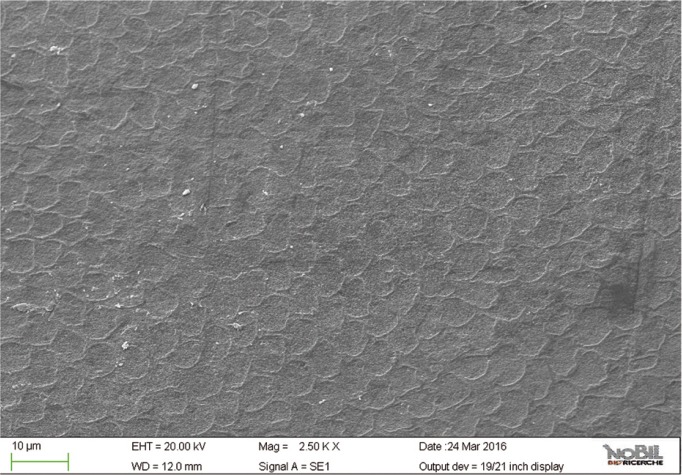
Means and standard deviations of the morphological SEM scores provided by the three observers.

**Figure 7:**
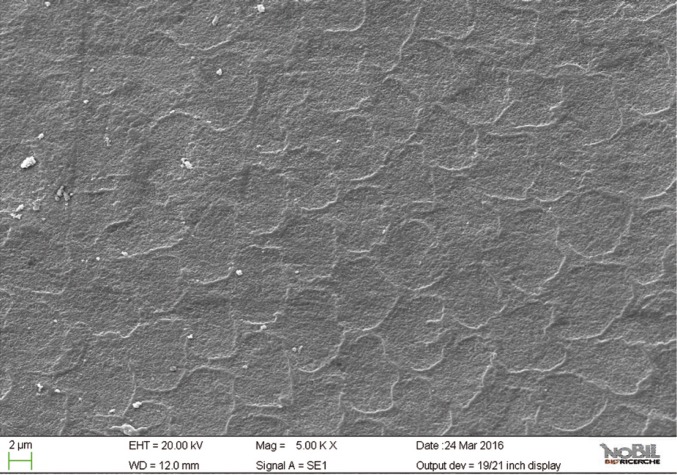
SEM image at 5.00 KX magnification of intact enamel surface treated with Subito (Group 3).

**Figure 8:**
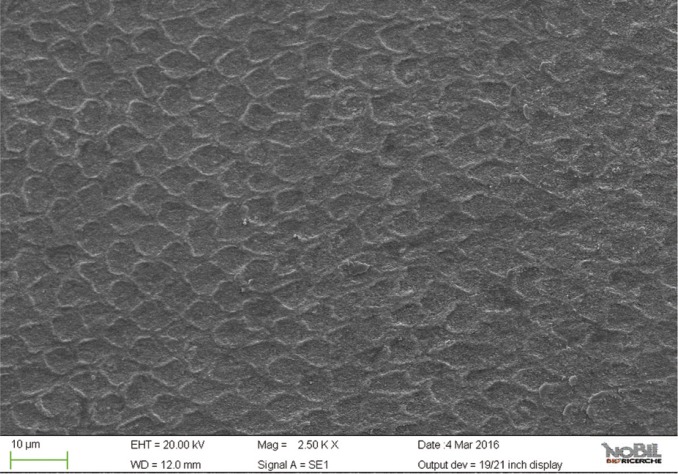
SEM image at 2.50 KX magnification of intact enamel surface treated with Eufresh (Group 4).

**Figure 9:**
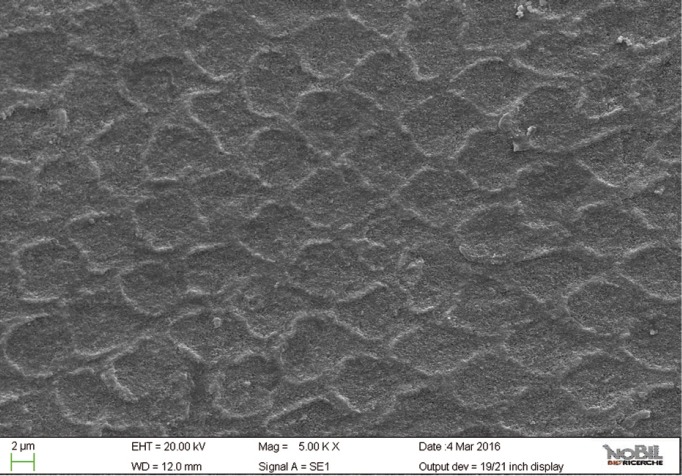
SEM image at 5.00 KX magnification of intact enamel surface treated with Eufresh (Group 4).

In the SEM images (Figs. 6-9) of the specimens treated with fluoride toothpaste (group 4) honeycomb structures that were typical of the demineralization enamel were still visible, and a slight irregular pattern of erosion could be observed. The average morphological score of this group was significantly lower than the scores for the acid challenged specimens (*p*<0.05).

The specimens treated with a non-fluoride toothpaste (group 3) showed intermediate values (*p*<0.05) between group 2 and 4, as confirmed by SEM images (Figs. 6,7); the morphological score assigned to group 3 is overall 2.48 ± 0.24.

Specimens of group 5 showed the lowest morphological SEM scores even if not as similar as intact enamel (*P*<0.05). Overall mean and standard deviations of the morphological SEM scores (1.3 ± 0.38) confirmed that Zn-HAP toothpaste provided the lowest evidence of erosive damage to the tooth surface (*p* < 0.001) among all groups exposed to the acid challenge and treated with the toothpastes.

**Figure 10:**
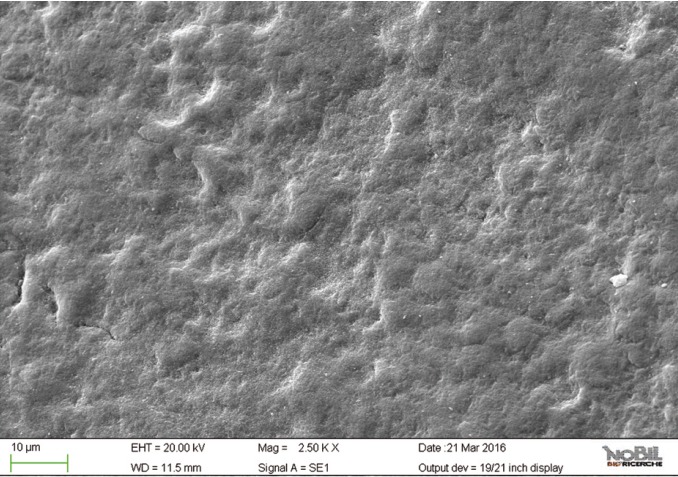
SEM image at 2.50 KX magnification of intact enamel surface treated with Biorepair (Group 5).

**Figure 11:**
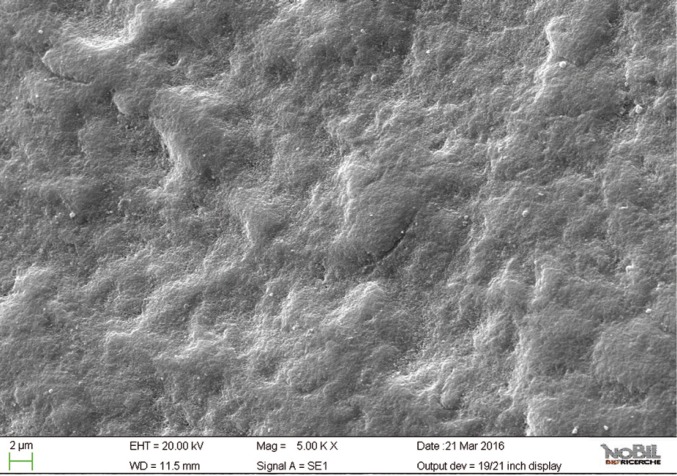
SEM image at 5.00 KX magnification of intact enamel surface treated with Biorepair (Group 5).

The specimens treated with the Zn-HAP toothpaste (group 5) showed evidence of deposited material in the SEM images (Figs. 10,11), with little evidence of erosive damage to the tooth surface.

Thus, if comparing the action of Zn-HAP (group 5), non-fluoride (group 3) and fluoride (group 4) toothpastes against an eroded enamel surface (group 2), it resulted that enamel specimens of group 5 tended to be significantly more protected after the treatment.

## Discussion

In this study the morphological analysis of enamel surfaces after an erosive acid challenge from a soft drink followed by treatment with Zn-Hap toothpaste showed a clear protective effect. This was greater than the effect observed for a normal fluoride toothpaste and for a toothpaste without fluoride and it confirmed the potential benefit the Zn-HAP technology can provide in protecting enamel from erosive acid challenges.

The morphological analysis of enamel was based on images taken by scanning electron microscopy (SEM), a technique that is suitable for use with native unpolished surface samples and enamel having been exposed to acidic challenge or toothpaste treatment. In the present *in vitro* study, SEM was used to verify the protective effect of the three toothpastes on enamel exposed to erosive action of a soft drink. The SEM study allowed to understand qualitatively the processes of demineralization of the enamel surface through the observation of specific morphological and structural features that characterize the enamel itself.

A classification scale was used in order to help quantifying and describe the damage grade on enamel. Scoring criteria modification of demineralization evaluation (16) was followed, as reported in  Table 2: a score of zero was assigned to enamel surface perfectly intact with no grooves, pits and porosity, while a score of three to those where diffuse demineralization involved the rod core, resulting in a lesion forming the “keyhole” like structure.

The experimental protocol of the present study was conducted in attempt to better simulate the daily habits of soft drink consumption. To predict the erosive potential of a soft drink, the method used should simulate what happens *in vivo* when the drink enters the mouth. For this reason, the method used in the present study (four consecutive intervals of 2 minutes for four times, at 0, 8, 24 and 36 hours) was considered to mimic, as closely as possible, the natural consumption of cola drink during the main daily meals. During the entire experimental protocol, the specimens were maintained in fresh artificial saliva until the next time of application of pastes. This means that the specimens were in contact with the bioactive agent for 12 min without suffering a demineralizing acid attack and then stayed in remineralizing solution.

In the oral environment, host factors (such as the mineral concentration of the tooth, and the pellicle and plaque formation) can influence the progression of demineralization (15,19). Salivary factors, such as the salivary flow rate, composition and buffering capacity, might exert protective action on dental surface (15,20,21).

Among soft drinks, Cola drink has the highest erosive potential (22,23) and this was the rationale for using it in the present study.

By comparing the SEM images of enamel treated with the Coca Cola to those of the unchallenged samples (Figs. 2-5), the remarkable effect of demineralization caused by the acidic drinks was significant. As expected, the surface of enamel treated with an acidic drink shows the presence of honeycomb structures, which suggests demineralization of enamel prisms. Diffuse demineralization involved the rod core, with decomposition of morphology of prims: they were severely affected and a greater prism-core dissolution compared with that in the interprismatic areas gave the enamel a “keyhole pattern” or “honeycomb pattern” of demineralization (Figs. 2,3).

In the present work, the protective efficacy of a Zn-HaP toothpaste was evaluated by SEM analysis: it was studied on enamel after acidic challenge and it was compared to a standard fluoride toothpaste, to a toothpaste without fluoride and to an untreated control. The results presented in  Table 3, supported by the images in figures 2-11 clearly demonstrated that the Zn-HAP technology was superior to other two toothpastes in protecting the enamel surface.

As expected, the highest score of damage was found in the samples challenged by the acidic drink and without toothpaste treatment, while the lowest degree of damage was recorded in the samples treated with Zn-HAP containing toothpaste. In the figures 4 and 5 (group 2) the enamel prism pattern showed a predominant dissolution of rods exposing interprismatic enamel. In the Figures 10 and 11 (group 5) the grade of damage observed in enamel surfaces after treatment with Zn-HAP dentifrice highlighted the persistence of rod integrity resembling a less advanced demineralization level if compared with samples treated with fluoride containing toothpastes and without fluoride (groups 4 and 3).

In the case of the Zn-HAP technology, this indicates that supplying calcium-phoshate minerals is a suitable and effective route to counteract the effect of an erosive challenge. The mode of action is a combination of reducing the demineralization effect of the acidic challenge and a remineralization/repair effect brought about by the extra provision of calcium and phosphates.
